# Annotating novel genes by integrating synthetic lethals and genomic information

**DOI:** 10.1186/1752-0509-2-3

**Published:** 2008-01-14

**Authors:** Daniel Schöner, Markus Kalisch, Christian Leisner, Lukas Meier, Marc Sohrmann, Mahamadou Faty, Yves Barral, Matthias Peter, Wilhelm Gruissem, Peter Bühlmann

**Affiliations:** 1 Institute of Plant Science, ETH Zurich, Universitaetsstr. 2, 8092 Zurich, Switzerland; 2 Seminar for Statistics, ETH Zurich, Leonhardstr. 27, 8092 Zurich, Switzerland; 3 Institute of Biochemistry, ETH Zurich, Schafmattstr. 18, 8093 Zurich, Switzerland; 4 Friedrich Miescher Institute, Maulbeerstrasse 66, Basel, Switzerland; 5 Competence Center for Systems Physiology and Metabolic Diseases (CC-SPMD), Zurich, Switzerland

## Abstract

**Background:**

Large scale screening for synthetic lethality serves as a common tool in yeast genetics to systematically search for genes that play a role in specific biological processes. Often the amounts of data resulting from a single large scale screen far exceed the capacities of experimental characterization of every identified target. Thus, there is need for computational tools that select promising candidate genes in order to reduce the number of follow-up experiments to a manageable size.

**Results:**

We analyze synthetic lethality data for *arp1 *and *jnm1*, two spindle migration genes, in order to identify novel members in this process. To this end, we use an unsupervised statistical method that integrates additional information from biological data sources, such as gene expression, phenotypic profiling, RNA degradation and sequence similarity. Different from existing methods that require large amounts of synthetic lethal data, our method merely relies on synthetic lethality information from two single screens. Using a Multivariate Gaussian Mixture Model, we determine the best subset of features that assign the target genes to two groups. The approach identifies a small group of genes as candidates involved in spindle migration. Experimental testing confirms the majority of our candidates and we present *she1 *(YBL031W) as a novel gene involved in spindle migration. We applied the statistical methodology also to TOR2 signaling as another example.

**Conclusion:**

We demonstrate the general use of Multivariate Gaussian Mixture Modeling for selecting candidate genes for experimental characterization from synthetic lethality data sets. For the given example, integration of different data sources contributes to the identification of genetic interaction partners of *arp1 *and *jnm1 *that play a role in the same biological process.

## Background

One of the major challenges in computational biology is the extraction of relevant information from the increasing amounts of data resulting from large scale experimentation. While the reliability of the results of single high-throughput assays has often been challenged, there is great promise that the confidence and precision of the outcomes can be increased through integration and combination of multiple data sources. We applied statistical modeling, based on data integration, for finding yeast genes involved in spindle migration from two synthetic lethality screens performed with *arp1 *and *jnm1*.

### Synthetic lethality data

Synthetic lethality describes the phenomenon of observing a lethal phenotype when two otherwise viable gene deletions are combined in one cell [1]. Since the yeast collection of deletion mutants has become available, genome-scale assays to uncover interactions between non-essential genes, for example by Synthetic Genetic Array (SGA) technology, have become routine in molecular genetics [2]. By using well-known genes as query genes and crossing them into the deletion set, one can systematically search for target genes that are synthetically lethal with the query gene. As a conclusion, these targets, together with the query gene, can be placed in a common functional context in the cell. Large scale synthetic lethal screens are a powerful genetics tool for studying vital biological processes and for finding new components involved in these processes. However, as with large scale experimentation in general, not all of the targets identified by such a screen are specific to the biological process that is investigated [3]. Two functionally very distant genes can show synthetic lethality because a gene deletion does not only cause the loss of function of a particular gene, but creates a whole cellular response to the loss of the gene, possibly affecting many pathways. As a a general example for synthetic lethality between two functionally distant genes, cell death might be the result of a disruption of a gene involved in DNA repair and another one with an important function in the primary metabolism. Since both genes act in processes that are relevant for cell survival it is more likely the general viability that is hampered than the functioning of a single or several interdependent processes. Due to crosstalk and partial redundancy between different pathways many genes that are found as targets in a synthetic lethal screen function in related but not directly associated biological processes. Thus in terms of pathway space a synthetic lethality screen performed by SGA yields close and distant genetic interaction partners of a given query gene. Of primary interest, however, are genetic interactions that occur within the same biological process because they indicate a close functional relationship between query and target gene.

Several approaches addressed the problem of characterizing synthetic lethal relationships.

Tong et al. applied hierarchical clustering to a set of 132 synthetic lethality screens to identify groups of genes acting in the same biological process [4]. They also found that, while synthetic lethal relationships are significantly enriched within protein complexes, members of a pathway show common genetic interaction partners rather than direct connections. Kelley and Ideker examined the topology of global genetic and protein networks with regard to the interaction structure [5]. After analysis of nearly 4800 synthetic lethal interactions and physical interactions involving close to 6000 proteins, the authors concluded that for a large part of known interaction data synthetic lethality can occur either within or between pathways with only a minority occurring within the same pathway. Using the same data set as Tong et al., work by Ye et al. showed that patterns of shared synthetic lethal interactions (rather than the genetic interactions themselves) are predictive of membership in a biological process [4,6]. Based on their analysis, they derived a congruence measure for assessing this correlation. They successfully validated their approach and proposed a novel gene acting in dynein-dependent spindle positioning. Applying the congruence score method on synthetic lethality data generated for 74 query genes involved in DNA damage response, Pan et al. identified 16 functional genetic modules that regulate DNA-damage checkpoint signaling and DNA repair [7].

All of the above reports suggest that synthetic lethal interactions which occur closely within the same biological scenario are a rare event. For an experimenter, however, they represent the interesting fraction of a synthetic lethal screen, especially when multiple sets of synthetic lethal data are not available for the pathway under investigation. Thus, the above mentioned methods are of limited utility. A close genetic interaction points to a target gene that can be readily examined by appropriate follow-up assays, whereas distant relationships remain difficult to tackle experimentally.

In the approach presented here, we focused on finding the subset of hits resulting from a synthetic lethality screen that is closely related to the query genes *arp1 *and *jnm1*. In contrast to the reports discussed above, our approach does not require large amounts of synthetic lethal information. Instead, we selected a list of genes identified as targets by two single synthetic lethal screens and investigated whether integrating additional genomic information could help to distinguish close genetic relationships from distant ones. Our approach complements previous conceptually different models that rely on large protein and/or genetic networks. Since we found a novel gene involved in spindle migration, and further provided functional annotation for an uncharacterized ORF, our success is on a comparable scale as described in [6].

In addition, we applied our statistical model to the biological process of TOR2 signaling and found that the predicted candidate genes are significantly enriched in a few known genes in the TOR2 signaling cascade.

### Our Approach

We analyzed the results of two synthetic lethal screens performed with *arp1 *and *jnm1*, two genes involved in the migration of the mitotic spindle [8,9]. This process is essential for high fidelity chromosome segregation and proper cell division in budding yeast. Here, it serves as a case study because some important regulatory elements are already known and can be used for reference (Table 1). In addition, spindle migration is a highly buffered process because of its importance for the cell. Both query genes used for the screens are involved in dynein-dependent spindle positioning. We chose *arp1 *and *jnm1 *as query genes, because an SGA-screen will detect genes functioning in pathways that compensate for the loss of dynein-dependent spindle positioning and thus also play a role in spindle migration. The screens were performed in the laboratory of Yves Barral and a majority of the hits found by Tong et al. were also identified in our screens (Additional File 1; [4]). For the study presented here, interactions from high-throughput synthetic lethal screening were not confirmed by tetrad assay in order to start the analysis with the largest possible candidate pool, including weak but potentially interesting phenotypes. Also, and more importantly, an adequate set of secondary assays is available for testing the outcomes of a computational analysis in an experimental setting.

**Table 1:** Genes in the data sets known to be related to spindle migration. For the statistical assessment (hypergeometric test) of the Gaussian Mixture Modeling results we used a reference set of all genes involved in spindle migration present in the data set analyzed. For some of the genes, their involvement in spindle migration has been published. The rest is known to be involved from the unpublished results of a previous Kar9-localization screen (Methods). Some key regulatory genes, such as *kar9 *and *bim1*, though being in the original synthetic lethality data set, are missing in the data set used for the analysis because of missing data for some observations (Additional File 5).

**Reference gene list**		
Gene name	ORF	Evidence

ELP6	YMR312W	unpublished data
BIM1	YER016W	[40]
YPT7	YML001W	unpublished data
PAT1	YCR077C	unpublished data
CCZ1	YBR131W	unpublished data
ASE1	YOR058C	[41]
TVP38	YKR088C	unpublished data

Since it is much easier to characterize genetic interactions between closely related genes in a follow-up experiment, that we pursued after statistical modeling, we concentrated our efforts on finding a subset of genetic interaction partners that have a close relationship to *arp1 *and *jnm1*, assuming that they also have a function in spindle migration.

Our method does not require a curated data set as a gold standard, as is the case in supervised learning approaches. Instead, it uses the structure found in the data for grouping in an unsupervised manner by way of a Gaussian Mixture Model. The parameters of the model are estimated by the Expectation Maximization (EM) algorithm.

**Figure 1:**
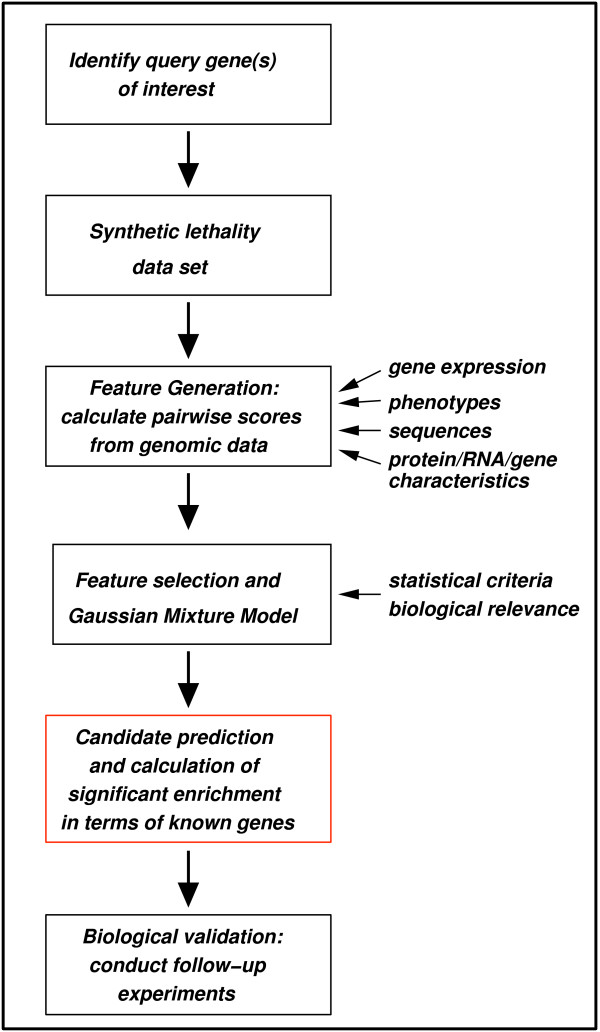
**Workflow chart of mixture modeling approach**. The figure depicts a general scheme of our mixture modeling method. First, one selects the query gene(s) from a biological process of interest. The respective synthetic lethal data set, retrieved either from a database, such as the GRID, or from own data then defines the list of target genes to consider [39]. Integration of different genomic information sources and generation of genomic features, that characterize the relationship of the query to its synthetic lethal targets, results in a multivariate data set of pairwise scores. Application of a Gaussian Mixture Model identifies a small group of target genes. Varying the posterior probability of the model refines the partitioning, such that the small group shows significant enrichment with known genes in the biological process of the query gene(s), if possible. As a last step, follow-up screens characterize the candidate genes contained in the small group in the biological context that is given (e.g. involvement in spindle migration).

To our knowledge, the presented method is novel because it uses mixture modeling as a framework for characterizing synthetic lethal interactions and for integrating different data types. Figure 1 shows a general outline of our approach. The following paragraphs describe how measurements of mRNA expression, phenotypic profiling, mRNA decay and sequence similarity were integrated and used for statistical modeling.

### Included Data Sets

When searching for target genes that are closely related to the query gene of an SGA, a protein interaction between both gene products is an obvious feature to consider. However, the protein interaction network measured on a large scale by two-hybrid or co-purification techniques only features the products of less than 10% of the genes in the data sets analyzed in this work. Hence, this incomplete information could not be included in a reasonable way in our model. Instead, we focused on data sources with good genome coverage to ensure that comprehensive information is incorporated.

Genes involved in the same biological process are likely to show similar mRNA-expression profiles [10]. To include knowledge about gene coexpression we chose three gene expression data sets. Gasch et al. tested 15 environmental and chemical stress conditions [11]. Hughes et al. measured changes in mRNA expression in response to 300 gene deletions and drugs [12]. Spellman et al. monitored the changes in mRNA expression at 80 experimental conditions related to the cell cycle [13]. For each of these data sets, we calculated the Pearson correlation coefficient of both query genes to all corresponding target genes. In the following, we will refer to these variables according to their source as *gasch.corr*, *hughes.corr *and *spellman.corr*, respectively.

In another microarray-based study, Brown et al. measured the sensitivity of the yeast gene deletion library to various growth conditions [14]. Strains responding to specific factors in the same or similar fashion are likely to carry deletions of genes that are functionally related [4,15]. We therefore calculated the Pearson correlation coefficients of the sensitivity profiles of all target genes to their corresponding query genes. We will refer to this variable as *pheno.corr*.

To include information about posttranscriptional regulation in the analysis, we considered the degradation rates of mRNA-transcripts. In a systematic approach, Wang et al. measured the mRNA decay rates of a comprehensive set of yeast ORFs [16]. The authors found similarities of transcript decay rates amongst genes encoding proteins of stochiometric complexes. To compare the rates of the different targets to the query genes we calculated the ratios of the respective decay rates and performed a log-transformation on them. We will refer to this variable as *logRNA.ratio*.

Proteins for similar biological or biochemical functions are likely to share common activity or structural domains. In fact, synthetic lethal interactions showed a significant enrichment among homologous gene pairs for functionally related proteins [4]. To include such information we determined the sequence similarities between the query genes and the corresponding target genes using blastp [17]. The log-transformed percentage values of sequence similarity were included in the analysis as the variable *logseq.sim*.

In total, we used 6 different data sources in addition to the information from synthetic lethal screens.

## Results

The statistical analysis performed on synthetic lethality data with query genes *arp1 *and *jnm1 *including the 6 additional data sources resulted in a small group of six genes that we propose to be closely related to *arp1 *and *jnm1 *and to have a function in the same biological process. The genes include *ase1*, *tvp38*, *uba4*, *gpd1*, *she1 *(YBL031W) a gene of unknown function, and the overlapping ORFs YHR127W and YHR131C, that we regard as one gene. Based on the analysis of a set of already known genes included in the data (Table 1 and Additional File 2), our group of closely related to *arp1 *and *jnm1 *is enriched for genes involved in spindle migration. Initial experimental validation of the candidates previously uncharacterized in this context confirmed an involvement for the majority of the genes in this statistically significant group. We provide functional annotation for YHR127W and *she1*, two genes whose exact function has been unknown.

### Feature selection

We computed every subset of variables derived from the six data sets (2^6^- 1 = 63) and used the Bayesian Information Criterion (BIC, Methods) and biological considerations to evaluate the sets of features (data sets) that approximate the data best and thus result in a reasonable model.

The variables *hughes.corr*, *spellman.corr *and *pheno.corr *were part of the top-scoring subsets, whereas subsets containing *gasch.corr*, *logRNA.ratio *and *logseq.sim *yielded worse results (data not shown). Thus, the variables *hughes.corr*, *spellman.corr *and *pheno.corr *contributed to a structure that allowed good separation of the data into two groups whereas the variables *gasch.corr*, *logRNA.ratio *and *logseq.sim *did not provide additional information. In a two-component Gaussian Mixture Model, the two components (two groups) stood for genes either having a direct involvement in spindle migration or no direct involvement.

**Figure 2:**
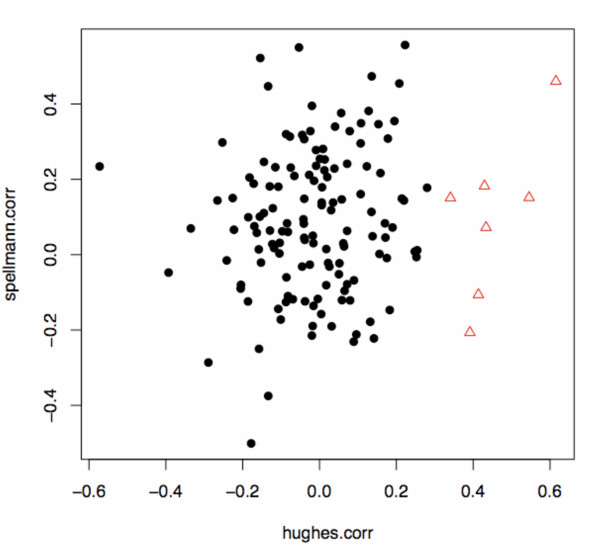
**Best subset of data sources**. Scatterplot for the best subset of features, which consists of *hughes.corr *and *spellman.corr*. The default separation is shown with a cutoff posterior probability of 0.5. The small group is shown in red triangles and the big group in black disks.

The best fit for two groups resulted from a using the variables *hughes.corr *and *spellman.corr *and thus only relied on transcriptional information. As illustrated in Figure 2, there is a small group consisting of seven data points (ORFs) corresponding to six different genes, that can be discerned from a bigger group containing the rest of the data. While the values of the variable *spellman.corr *do not differ much between the two groups, the variable *hughes.corr *is indicative of separation.

All members of the small group share intermediate to high values of correlation (0.3–0.65) in the *hughes.corr*-data set as opposed to the members of the larger group that fall in a range between -0.4 and 0.3. Although, only the information of *hughes.corr *seemed to be important at first sight, the more detailed statistical analysis shows that this is not the case: The use of *hughes.corr *and *spellman.corr *was clearly more suitable with respect to the BIC score.

### Statistical Assessment

Given unequal grouping, one would naturally consider the small group to comprise interesting information. Also, as already pointed out in the introduction, only a minority of the genes with synthetic lethal interactions are closely related [2,4-6]. We would therefore expect the small group to contain genes with close functional relationships. In line with these prior speculations, we found a small group of genetic interaction partners in the data that differentiate themselves from the larger remainder of the data, because they belong to a different multivariate Gaussian component. Given unequal grouping the smaller group intuitively can be considered specific as opposed to the big remainder of unspecific data points. The high positive correlation values for the variable *hughes.corr *showed that these target genes are transcriptionally co-regulated with either *arp1 *or *jnm1 *and further suggested direct involvement in the same biological process. Thus, they represent promising candidates for thorough experimental testing. For the grouping illustrated in Figure 2 the default cutoff for the posterior probability in the Gaussian Mixture Model was set to 0.5 to separate the small from the large group (Additional Files 3 and 4). Genes with a higher posterior probability were assigned to the small group and genes with lower values comprised the large group. Variation of this threshold shifts the quantitative proportion between both groups. To judge the enrichment of the small group with genetic interaction partners known to be involved in spindle migration, we used a reference list of seven out of the 129 genes contained in the data set that are known to be involved in spindle migration (Table 1 and Additional File 5). Employing a hypergeometric test showed the number of known genes in the smaller group from our model to be significantly higher than would be expected by chance. Furthermore, we analyzed the enrichment when changing the size of the small group. We reduced the cutoff for the posterior probability so that the small group contained 50 genes. In both cases, the small group showed a significant enrichment of known spindle migration genes (Table 2).

**Table 2:** P-values for best model for spindle migration. Statistical assessment of the best subset of features {*hughes.corr*, *spellman.corr*}. The p-values based on the hypergeometric test are shown for two different group sizes. In addition the numbers of known genes and the total number of genes in the data set.

**P-values for small group**				
Genes in small group	Known genes	Known genes in data set	Genes in data set	P-value

6	2	7	129	0.0342
50	6	7	129	0.0136

The lists of genes resulting from both small groups (of either size 6 or 50) can be considered as promising candidates for further biological experimentation. They are significantly enriched in genes known to be specific for spindle migration and thus it is likely that among the rest of the genes in these lists, which are unknown with respect to spindle migration, additional members of this biological process can be found.

### Experimental Validation

In order to biologically validate the six candidates in the small group (Table 3) we reviewed what was known about the genes in the literature and also conducted experiments using time-lapse microscopy (Methods). Proper spindle migration requires several cellular processes such as spindle integrity, spindle elongation and localization of Kar9. Thus, the genes in the list cannot be expected to show a unique phenotype that could be detected by a single assay. Therefore, we tested for perturbations in any of these processes.

**Table 3:** Experimental Validation. Phenotypes of the 6 members of the small group.

**Experimental validation of small group members**		
Gene name	ORF name	Experimental Evidence

ASE1	YOR058C	compromised anaphase spindles
TVP38	YKR088C	perturbed Kar9-asymmetry
SHE1	YBL031W	broken spindle; perturbed Kar9-asymmetry
UBA4	YHR111W	perturbed Kar9-asymmetry
YHR127W	YHR127W	weakly perturbed Kar9-asymmetry
GPD1	YDL022W	none detected

The two genes, *ase1 *and *tvp38 *had previously been described in the context of spindle migration. *ase1 *had already been reported to be required for spindle integrity. In a deletion strain, the spindles fail to elongate during anaphase and Ase1 localizes to the spindle midzone, indicating that Ase1 is required for high fidelity chromosome segregation [18,19]. In another assay, asymmetric localization of Kar9 is used to identify genes with a function in spindle migration [20].

**Figure 3:**
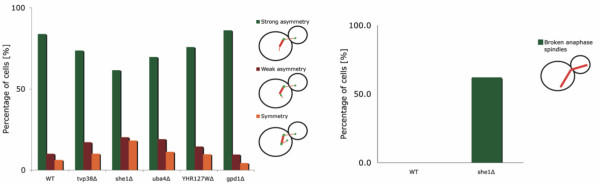
**Experimental validation of genes predicted to be involved in spindle migration**. A) The *she1*Δ, *uba4*Δ and YHR127WΔ-strains show perturbed Kar9 localization. *gpd1*Δ does not show a phenotypic effect. Wild type and *tvp38*Δ are shown as positive and negative controls, respectively. The differences in proportions of defective cells are compared to the wild type based on the binomial distribution. This results in p-values that are significant for *she1*Δ and *uba4*Δ (p-values 0.0008% and 0.28% respectively), marginally significant for YHR127WΔ (p = 7.4%) and not significant for *gpd1*Δ (p = 70%). B) Some *she1*Δ cells have broken or bent anaphase spindles suggesting compromised spindle integrity.

¿From previous experiments, it was known that *tvp38*Δ cells show perturbed asymmetric Kar9 localization. Only 73.6% of the *tvp38*Δ cells have Kar9 localized in a strongly asymmetric manner as opposed to 83.8% in WT cells (Figure 3, Additional Files 6, 7, 8). This indicates that Tvp38 plays a role in maintaining Kar9 asymmetry. For the reasons mentioned above, we considered both genes as required components for spindle migration and used them in the reference gene list for statistical assessment. The additional genes found in the small group of six genes were analyzed by the same experimental methods.

The *gpd1*Δ mutant strain showed no phenotype. *uba4*Δ-strain another partially redundant urmylation enzyme is active. The strain carrying a deletion of the uncharacterized ORF YHR127W, showed a Kar9 localization phenotype similar to that seen in *tvp38*Δ cells, although the phenotype was less pronounced (75.8% asymmetry vs. 83.8% in WT, Figure 3 and Additional File 8 and 9). For *uba4*Δ a strong Kar9-localization phenotype was detected (69.7% asymmetry, Figure 3 and Additional File 8 and 10). Uba4 is the enzyme required for post-translational regulation via urmylation [21]. Deletion of *she1 *(YBL031W), which encodes a cytoskeletal protein the exact function of which has remained elusive, resulted in a marked decrease in Kar9 asymmetry as well as a high frequency of broken spindles in anaphase cells (61.6% asymmetry, Figure 3, Additional Files 8 and 11, 12, 13). This gene displayed a phenotype in both assays used for validation, which provides strong support for an involvement in spindle migration. These results demonstrate that most of the six genes in the small group of our statistical model have a role in spindle migration. Only for one of the genes, *gpd1*, no direct experimental proof was found. The function of *ase1 *and *tvp38 *in spindle migration has been known beforehand. The phenotype of YHR127W, being relatively mild, suggests a moderate effect of this gene on spindle migration. For the other two genes, *uba4 *and *she1*, strong evidence exists that they have a direct role in the proper functioning of spindle migration and chromosome segregation. Interestingly, the role of YHR127W and *she1 *in this context is a novel finding and clear function annotation has still been missing for both genes. Finding a kar9-localization defect for *uba4*Δ is consistent with the same phenotype seen after deleting its direct target in urmylation, *urm1*, which was assessed in a previous screen (Additional File 2). This strongly points towards an effect of protein-urmylation on spindle migration. In conclusion, three of the additionally found candidates deserve further experimental characterization in order to determine their mechanistic involvement in spindle migration more precisely.

### Application to TOR2 signaling

To evaluate the general usefulness of the statistical method, we considered TOR2 signaling as another biological process. Apart from a shared function with its homolog *tor1*, *tor2 *regulates the spatial organization of cell growth, mainly through actin cytoskeleton polarization [22]. Using the same approach as described above for spindle migration we aimed to find additional genes involved in the specific function of *tor2*, which we term TOR2 signaling. Since *tor2 *is an essential gene, we analyzed data from a synthetic lethal screen performed with the ts-mutant *tor2-21 *(Additional File 14). We considered a set of seven input variables (Additional File 15), most of which were different from the ones used in the analysis for spindle migration. Since mRNA decay rates, sequence similarity, phenotypic profiling and the Gasch et al. gene expression data were not meaningful in the first study, they were replaced with other features. Instead of the cell cycle gene expression data from Spellman et al. we included the data from Mnaimneh et al., a larger compendium of gene expression profiles [23]. The following paragraphs describe the list of features. crude measure and the use of this feature for gene pairs lying on different chromosomes is unclear. For genomic loci on the same chromosome, though, the feature can contain important information.

In a recent study Warringer et al. investigated the influence of various biological features on protein size [24]. While they found a correlation between protein size and gene expression as well as biochemical activity no effect was found for pleiotropy of genes, for instance. To assess whether we find similarities in protein size between *tor2 *and its genetic interaction partners we considered molecular weight as a proxy and calculated the absolute log ratios for the corresponding protein products of *tor2 *and its targets, resulting in the input feature *MW.ratio*.

Sharp et al. have shown that some codons are optimal for expression of a gene, because the respective tRNAs are most abundant [25]. They formulated the codon adaptation index (CAI), a score that assesses the relative occurrence of codons in a gene that are optimal for its expression. Since similar codon bias can point to close functional relationships between two genes, we calculated the absolute difference between the CAI index values for *tor2 *and its targets and named the input feature *CAI.diff*.

Similarities in the relative frequency of amino acids in two proteins can suggest common molecular functions and point towards close relationships between genes encoding them. For instance, two gene products containing many hydrophobic residues are both likely membrane proteins. The GRAVY score measures the hydropathicity of a protein, while the AROMA score assesses the relative content of aromatic amino acids in a protein [26,27]. Tor2 is known to localize to membranes, thus especially the GRAVY score is an interesting feature to consider. For the input features *GRAVY.diff *and *AROMA.diff*, we calculated the absolute difference of theses scores for *tor2 *and its targets.

For the above features small scores stand for close functional similarity between the respective genes. As another sequence based input feature we assessed correlations in total amino acid content. Different studies have shown the influence of biological characteristics of proteins on their amino acid composition [28,29]. We exploit similarities of gene products in amino acid content in order to infer functional relationships between them. Hence, we calculated the Pearson correlation coefficient of the amino acid profile (i.e. how often each amino acid occurs in the protein) corresponding to *tor2 *and its targets. This resulted in the variable *aa*.

As in the first analysis for spindle migration, we also considered gene expression data for the generation of input features. For correlations in gene expression data sets we calculated the Pearson correlation coefficient of the gene expression profiles of *tor2 *and its targets in the Hughes et al. and the Mnaimneh et al. data sets [12,23]. The first one was already described in the analysis of spindle migration. In the study of Mnaimneh et al. gene expression was measured for mutant strains carrying titrable promoter alleles of all essential genes. We included the corresponding features under the names *hughes *and *mnaimneh *in the analysis. The data for the features *CAI.diff*, *GRAVY.diff*, *AROMA.diff*, *MW.ratio *and *aa *were downloaded from SGD [30].

Following the same strategy as for the first analysis, we selected the three input variables *aa*, *hughes *and *mnaimneh*, which resulted in clearly unequal grouping. As described for the modeling of spindle migration, we used the default cutoff posterior probability of 0.5 to identify the small group (Additional File 16). For further analysis of the members of this group we used a reference list of four genes, known to be specific to the biological process under investigation. The products of *avo2 *and *slm1 *are subunits TORC2, the multi-protein complex containing Tor2 [31,32]. *bck1 *and *slt2 *encode protein kinases acting in the MAP kinase pathway mediated by protein kinase C, which is downstream of *tor2 *[33]. Thus all of those 4 genes are involved in TOR2 signaling. With a default cutoff of 0.5 no significant enrichment was found for the small group. As in the first analysis for the spindle migration data, we subsequently lowered the posterior probability to 0.29 which resulted in a small group of 18 candidates out of a total of 70 genes in the data set that were enriched in genes with a direct role in TOR2 signaling (Additional File 17 and Table 4). The scatterplot shows that the identified small group is characterized by very high values of *aa *as well as intermediate to high values of *hughes *and *mnaimneh *(Additional File 18). Thus, the genes in the small group are positively correlated with *tor2 *in terms of their amino acid content and their expression profiles in the Hughes et al. and the Mnaimneh et al. studies. Thus, also for TOR2 signaling, our statistical method resulted in a list of target genes with good prospects for further experimentation.

**Table 4:** P-values for best model for TOR2 signaling. Statistical assessment of the best subset of features (Additional File 17 and 18). The p-value based on the hypergeometric test is shown for group size 18. The table presents the numbers of known genes and the total number of genes in the data set.

**P-values for small group**				
Genes in small group	Known genes in small group	Known genes in data set	Genes in data set	P-value

18	3	4	70	0.0496

This demonstrates that our approach has broader utility and can be applied to other synthetic lethality data sets. It suggests that the statistical method has a general potential of finding new genes in a biological process.

## Discussion

We used and evaluated an unsupervised statistical method that relies on data integration for separating synthetic lethal interaction partners of *arp1 *and *jnm1 *into those that are specific to spindle migration and those representing the unspecific or more distantly related remainder of genetic interaction partners. Multivariate Gaussian Mixture Modeling was applied to divide different subsets of a heterogeneous genomic data set into two groups. The two features *hughes.corr *and *spellman.corr *derived from two gene expression data sets resulted in the best model fit. For this subset, we identified a small group of six genes that was significantly enriched in known spindle migration genes. Moreover, biological testing of the top scoring genes that had been uncharacterized in this context (two genes had been characterized already) yielded experimental confirmation for *she1*, *uba4 *and YHR127W as being involved in spindle migration. In a modeling analysis for *tor2 *we also identified a small group of candidate genes with significant enrichment in terms of known genes in TOR2 signaling.

For both analyses it is not suprising that BIC selects a model only consisting of a subset of all inputs. BIC is designed to penalize input variables that do not contain useful information (Methods). For the example of the spindle migration analysis this means that all other inputs are uninformative when having *hughes.corr *and *spellman.corr *in the model. For the TOR2 analysis all other features are uninformative when *aa*, *hughes *and *mnaimneh *are present in the model. There are different reasons why features can be uninformative in the context of a two-component Gaussian Mixture model. i) The means and the covariances of the uninformative features are (approximately) the same for both groups; ii) there are high (partial) correlations between the uninformative inputs and the features which are already in the model; or iii) a distinction into two components with many parameters (due to too many features) can become inaccurate given limited sample size. We demonstrate the usefulness of this feature selection approach by presenting biologically meaningful results. The finding that models with few strong features are powerful has also been reported in other work not relying on the BIC for feature selection [34,35]. This shows that feature reduction is important in data integration approaches.

In the analysis for the spindle migration genes, correlating mRNA-expression is more informative of a close mutual relationship than is comparing their sequence similarity or their rates of mRNA-decay. Here, transcriptional information helps to identify close genetic interaction partners of the query genes *arp1 *and *jnm1*, while the other variables don't contribute to the model (worse BIC scores when included). Also for TOR2 signaling, gene expression correlations in addition to similarity in amino acid composition was important for the identification of the close genetic interaction partners. The selected group of candidate genes all share intermediate to high correlations in their gene expression profiles in the Hughes et al. and Mnaimneh et al. data sets and have very similar amino acid content. correlations give preliminary biological support for the genes to be closely related to *tor2*. This together with the enrichment of genes known to function in TOR2 signaling strongly suggests the selected list of genes to represent good candidates for further experimental testing, since they are likely to have a close functional relationship to *tor2*.

It is one of the main advantages of the model that it is flexible with respect to the input features and that it identifies the most relevant subset of inputs for the specific biological process under investigation. This stands in contrast to other data integration approaches that globally combine large information resources for predictions, such as the STRING database or bioPIXIE [36,37].

Our approach is conceptually novel and different from existing approaches for synthetic lethal data analysis. Current methods rely on a substantial number of screens or global synthetic lethality data found in databases [4-7]. Thus, they are suited for pathway analysis on a global level, when the biological process of interest has been extensively studied by genetic interaction screens. The Gaussian Mixture Model proposed here is advantageous in situations with incomplete data, for weak phenotypes in a synthetic lethal screen and for characterizing synthetic lethal interactions. This is the case when studying yeast pathways that have not been explored extensively by synthetic lethality screening, so that only a handful of data sets exist. Or studies in other organisms, such as *Caenorhabditis elegans *or *Drosophila melanogaster*, where synthetic lethal screens are being carried out, but to a much smaller extent than in yeast.

For the example of spindle migration, our approach successfully identified novel gene functions. Since most of the candidates identified in the top 6 of our model actually play a role in spindle migration, we presume that among the top 50 genes whose respective p-value is actually smaller than for the top 6 (Table 2) many genes function directly in spindle migration.

Yet, there are a few limitations to our approach: Since our analysis is based on just two synthetic lethal screens, not all known spindle migration genes could be found. Essential genes and genes from the dynein-dependent spindle positioning pathway are missing in the analysis since they could not be detected by synthetic lethality, others, such as *kar9*, had to be excluded from the analysis due to missing data. Further, one has to keep in mind, that genes showing high similarities with the two query genes *arp1 *and *jnm1 *do not necessarily have to be related to spindle migration since both genes are involved in several processes where movement of microtubules is required, such as cell polarity, cell migration, vesicle transport, and the formation of membrane protrusions. Nevertheless, we focused on their role in spindle migration because it is the function that is experimentally described best, and because suitable assays for testing promising genes in that context were available. Indeed, experimental validation of a small group of candidate genes has supported the model-based predictions and adds initial biological evidence to the assumption that most of these genes are involved in the process that was studied. Still, we provide experimental support for the genes *she1 *and YHR127W to play a role in spindle migration. The fact that we obtained novel findings for the function of these two genes in spindle migration clearly demonstrates the usefulness of our approach. Moreover, we propose the urmylation enzyme *uba4 *as another gene involved in spindle migration, a finding that is in agreement with the previously found involvement of *urm1 *and strongly suggests the requirement of urmylation for this process.

Application of the same statistical methodology on synthetic lethality data for a *tor2 *ts-mutant using a different set of genomic features also resulted in lists of new candidate genes potentially involved in TOR2 signaling. The method is thus of general utility to find potential candidate genes from synthetic lethal screens.

## Conclusion

The presented work shows that a Multivariate Gaussian Mixture Model as a framework for data-integration is suitable for the analysis and characterization of synthetic lethality data. We demonstrate an efficient way, in terms of a statistical model, to reduce the list of target genes from a screen to a set of candidates with good prospects for further experimentation in the laboratory. Since the amounts and the quality of high-throughput data will increase in the future, more and better biological features can be expected to arise. Including them in our model will increase its predictive power and accuracy.

## Methods

### Mixture modeling

We assume that the gene pairs exhibiting synthetic lethality with *arp1 *and *jnm1 *(synthetic lethality interactions) can be divided into two groups, one of them representing genes involved in spindle migration and the other representing genes with no direct involvement. We model this group membership with a latent (unobserved) variable *Z *with values in {1, 2}. The goal is to infer the latent states (the group memberships) from data. To do so, we assume the following model: the conditional distribution of the data **x**_**i**_∈ *R*^6 ^(e.g. the 6-dimensional observation from the 6 genomic data sets for the spindle migration analysis, i.e. *gasch.corr, hughes.corr, spellman.corr, pheno.corr, logRNA.ratio, logseq.sim*, for every gene pair *i*) given the state of the latent variable is multivariate (6-dimensional) Gaussian with mean vector *μ*_*k *_and covariance matrix Σ_*k *_if *Z*_*i *_= *k *(*k *= 1, 2). By using Bayes' Theorem, we can infer the posterior probabilities *P*[*Z*_*i *_= *k|x*_*i*_] (*k *= 1, 2) which yield a probabilistic prediction for the *i*th gene-pair belonging to group 1 or 2.

The model we just described is a Gaussian Mixture Model (GMM) with 2 groups (2 states for the latent variable). Its probability density takes the form:

$$f(x|\mu_{1},\Sigma_{1},\mu_{2},\Sigma_{2})=\sum_{k=1}^{2}\pi_{k}\phi (x|\mu_{k},\Sigma_{k})$$

where *φ*(**x ***|μ*_*k*_, *Σ*_*k*_) is the probability density of the multivariate Gaussian distribution corresponding to group *k*:

$$\phi (x|\mu_{k},\Sigma_{k})={\left(2\pi \right)}^{-\frac{p}{2}}\det{\left(\Sigma_{k}\right)}^{-\frac{1}{2}}\exp\left(-\frac{1}{2}{\left(x-\mu_{k}\right)}^{T}\Sigma_{k}^{-1}(x-\mu_{k})\right)$$

The parameters can be found by the EM algorithm. The optimal set of variables of other data sources is the one resulting in a minimal Bayesian Information Criterion (BIC), which is defined as:

*BIC *= -2 ln (*L*) + *d *ln (*n*),

where ln(*L*) is the log likelihood, *d *is the number of parameters and *n *is the sample size. We used the R-package mclust [38] for all calculations.

All samples can then be assigned to one out of the two groups by inspecting the posterior probabilities *P*[Group = *k |***x**], *k *∈ {1, 2}. As mentioned above, the sample *i *(gene-pair *i*) can be assigned to one of the two groups by inspecting the posterior probability *P*[*Group *= *k|x*_*i*_] = *P*[*Z*_*i *_= *k|x*_*i*_] (*k *= 1, 2).

Of course, other approaches for unsupervised assignment of a sample (gene-pair) to a group could be used as there exists a wide variety of clustering methods. We prefer to work with a model, i.e. a GMM, rather than just an algorithm. Due to the fact that we work with a model, the GMM approach easily allows: (i) to do feature selection (selection of genomic data sets) via the BIC criterion; (ii) to assign interpretable probabilities for group-membership; (iii) for additional flexibility to select the number of groups (in our case equal to 2) via the BIC criterion. Finally, the most natural assumption on the distribution of the data given the latent variable *Z *(see description above) is the multivariate Gaussianity.

### Experimental Procedures

#### SGA

Performed as described in [2]. In brief, query strains used were, arp1::kanMX his3 leu2 ura3 lys2 can1::MFA1pr-HIS3 and jnm1::kanMX his3 leu2 ura3 lys2 can1::MFA1pr-HIS3. Each strain was crossed into the Yeast Knock Out Collection (Open Biosystems), and double knock-out strains were scored for synthetic lethality. Each screen was performed once.

#### Yeast Strains

Deletion strains were generated for each predicted ORF in the presence of a chromosomal CFP-tub1:URA marker in the URA3 locus and a chromosomal kar9-YFP:NAT marker in the endogenous kar9 locus. Integration of CFP-tub1 and C-terminal tagging with YFP was done as described in (Liakopoulos et al., 2003).

#### Time-Lapse Fluorescence Microscopy

Fluorescence microscopy was performed on an Olympus BX50 fluorescence microscope equipped with a piezo motor, Polychrom IV monochromator as light source, a high speed CCD camera (Imago, TillPhotonics) and TILLVision software (TILLPhotonics, Martinsried, Germany). Dual color acquisitions were performed using a Chroma CFP/YFP dual band filter.

Images were acquired as stacks of 5 focal slices, 0.4 *μ*m between each slice. Each time-lapse series was recorded with 10 time frames and presented as 10 maximum projections with 10 s intervals over the course of 100 s. The time-lapse series were acquired in a YFP and a CFP channel and fused as RGB movies in NIH Image J.

#### Spindle Integrity and Elongation

Cells with reduced spindle integrity may have difficulty with spindle elongation during anaphase [18,19]. We quantified the fraction of WT vs. Δ cells with compromised anaphase spindle integrity. Breaking and bending of the spindle was scored. The experiment was performed with two independent clones per strain, and n > 20 anaphase cells per strain. For single cell illustrations see supplement.

#### Kar9 Localization

Localization of spindle positioning protein Kar9 on the spindle pole body and the astral microtubules occurs only on the bud proximal side of the spindle. This asymmetric localization of Kar9 is essential for proper function of spindle migration. We scored for asymmetric Kar9 localization in all the predicted mutants. We quantified the fraction of cells with Kar9 localized in a strongly asymmetric, weakly asymmetric and symmetric manner. The experiment was performed with two independent clones per strain (n ≈ 100 cells). For single cell illustrations see supplement.

#### Yeast strains used in this study

**KAR9::YFP:NAT ura3::TUB1-CFP:URA3 gpd1::kanMX **ade2-101ura3-52 lys2-801 his3-Δ200 trp1-Δ63 leu2

**KAR9::YFP:NAT ura3::TUB1-CFP:URA3 she1::kanMX **ade2-101ura3-52 lys2-801 his3-Δ200 trp1-Δ63 leu2

**KAR9::YFP:NAT ura3::TUB1-CFP:URA3 tvp38::kanMX **ade2-101ura3-52 lys2-801 his3-Δ200 trp1-Δ63 leu2

**KAR9::YFP:NAT ura3::TUB1-CFP:URA3 YHR127W::kanMX **ade2-101ura3-52 lys2-801 his3-Δ200 trp1-Δ63 leu2

**KAR9::YFP:NAT ura3::TUB1-CFP:URA3 **ade2-101ura3-52 lys2-801 his3-Δ200 trp1-Δ63 leu2

## Abbreviations

BIC – Bayesian Information Criterion

CAI – Codon adaptation index

EM algorithm – Expectation Maximization algorithm

GMM – Gaussian Mixture Model

ORF – Open reading frame SGA – Synthetic Genetic Array

ts-mutant – temperature sensitive mutant

## Authors' contributions

DS, MK, CL and LM contributed equally to the work presented in this manuscript. DS, MK and LM performed the statistical analyses, CL carried out the experiments. All authors were involved in the design of the study and in discussions about the work. All authors have read and approved the manuscript.

## Supplementary Material

Additional File 1 **Synthetic lethality data for spindle migration genes**. The xls-file contains the standard and systematic gene names for all synthetic lethality interactions found in a systematic screen performed with *arp1 *and *jnm1 *performed in the lab of Yves Barral. Click here for file

Additional File 2 **Genes known to be involved in spindle migration**. The xls-file contains all genes that were experimentally shown to be involved in the process of spindle migration. The list covers genes of the dynein-dependent pathway, the Kar9-dependent pathway and genes that showed defects in Kar9-localization. Click here for file

Additional File 3 **Histogram of the posterior probabilities for the small group of the spindle migration mixture model**. The pdf-file contains a figure showing the distribution of posterior probabilities for the small group of genetic interaction partners with *arp1 *and *jnm1*. High posteriors mean high likelihood for the respective genes of being involved in spindle migration. Click here for file

Additional File 4 **List of genes ranked according to their probability for involvement in spindle migration**. The xls-file contains all genes used in the mixture modeling approach in the order of their probability of being involved in spindle migration. The genes already known to be involved are marked in cyan. The top 6 and top 50 groups are marked with a black horizontal line. Click here for file

Additional File 5 **Data matrix used for mixture modeling for spindle migration**. The xls-file contains the data set that was used for mixture modeling for the *arp1 *and *jnm1 *targets. Due to missing data in the source data sets biological variables were only calculated for genetic interactions for which complete information was available. Click here for file

Additional File 6 **Movie of Kar9-localization in WT cell**. Avi-file showing WT cell in metaphase. The cell is expressing CFP-Tub1 (red) and Kar9-YFP (green). 10 images were captured every 10s showing Kar9 localizing on the SPB and astral MT on bud-directed pole only (asymmetric). Click here for file

Additional File 7 **Movie of Kar9-localization in a tvp38Δ cell**. Avi-file showing a *tvp38*Δ cell in metaphase. The cell is expressing CFP-Tub1 (red) and Kar9-YFP (green). 10 images were captured every 10s showing Kar9 localizing on the SPB and astral MT on both sides of the spindle (symmetric). Click here for file

Additional File 8 **Quantification of Kar9-localization**. Xls-file showing the quantified results for the experimental validation based on the Kar9-localization phenotypic screens. The results for each gene in the small group are shown for two separate clones and averaged over both clones. Click here for file

Additional File 9 **Movie of Kar9-localization in a YHR127WΔ cell**. Avi-file showing a YHR127WΔ cell in metaphase. The experiment was carried out as for *tvp38*Δ and revealed a similar phenotype. Click here for file

Additional File 10 **Movie of Kar9-localization in a uba4Δ cell**. Avi-file showing a *uba4*Δ cell in metaphase. The experiment was carried out as for *tvp38*Δ and revealed a similar phenotype. Click here for file

Additional File 11 **Movie of Kar9-localization in a she1Δ cell**. Avi-file showing a *she1*Δ cell in metaphase. The experiment was carried out as for *tvp38*Δ and revealed a similar phenotype. Click here for file

Additional File 12 **Movie of spindle integrity and elongation in WT cell**. Avi-file showing a WT cell in anaphase. The cell is expressing CFP-Tub1, which labels the elongated anaphase spindle. 10 images were captured every 10s revealing the relatively rigid structure of the spindle. Click here for file

Additional File 13 **Movie of spindle integrity and elongation in mutant cell**. Avi-file showing a *she1*Δ cell in anaphase. The cell is expressing CFP-Tub, which labels the elongated anaphase spindle. 10 images were captured every 10s showing the spindle breaking due to loss of spindle integrity in this mutant. Click here for file

Additional File 14 **Synthetic lethality data for TOR2 signaling**. The xls-file contains the standard and systematic gene names for all synthetic lethality interactions found in a systematic screen performed with *tor2-21 *performed in the lab of Charlie Boone. The screen was performed twice and only hits identified in both rounds are in the data set. Click here for file

Additional File 15 **Data matrix used for mixture modeling for TOR2 signaling**. The xls-file contains the data set that was used for mixture modeling for the *tor2-21 *targets. Due to missing data in the source data sets biological variables were only calculated for genetic interactions where complete information was available. Click here for file

Additional File 16 **Histogram of the posterior probabilities for the small group of the TOR2 signaling mixture model**. The pdf-file contains a figure showing the distribution of posterior probabilities for the small group of *tor2 *genetic interaction partners. High posteriors mean high likelihood for the respective genes of being involved in TOR2 signaling. Click here for file

Additional File 17 **List of genes ranked according to the posterior probability for involvement in TOR2 signaling**. The xls-file contains all genes used in the mixture modeling approach in the order of their probability of being involved in TOR2 signaling. The genes already known to be involved are marked in yellow and the literature reference is given in the file. The top 18 is marked with a black horizontal line. Click here for file

Additional File 18 **Best subset of features for TOR2 signaling**. The pdf-file shows the scatterplot for the best subset of features for the TOR2 signaling mixture model {*aa*, *hughes*, *mnaimneh*} with a cutoff of 0.29. Target genes belonging to the small group are marked by red triangles, the big group is marked in black. The members of the small group all share very high values for amino acid correlation (*aa*) and intermediate to high values of correlation in gene expression data with respect to *tor2 *(*hughes *and *mnaimneh*). Click here for file
