# Supplementary figures and images for: Annotating novel genes by integrating synthetic lethals and genomic information
Source: BMC Syst Biol. 2008 Jan 14;2:3. doi: 10.1186/1752-0509-2-3 (PMC2258006; doi:10.1186/1752-0509-2-3)

## Grouping for spindle migration

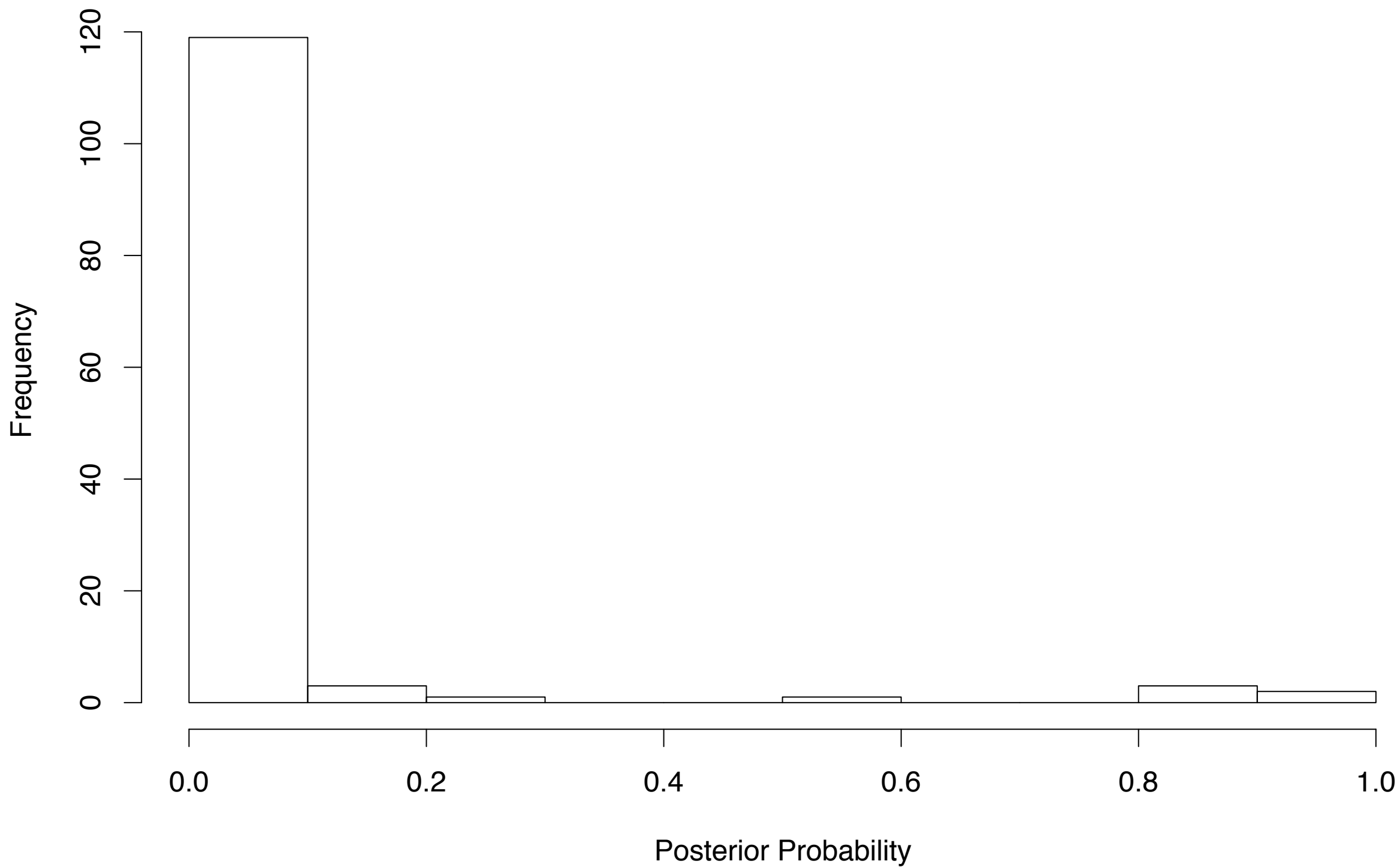

Supplement: Additional File 3 — Histogram of the posterior probabilities for the small group of the spindle migration mixture model. The pdf-file contains a figure showing the distribution of posterior probabilities for the small group of genetic interaction partners with arp1 and jnm1. High posteriors mean high likelihood for the respective genes of being involved in spindle migration. [file 1752-0509-2-3-S3.pdf]

## Grouping for TOR2 signaling

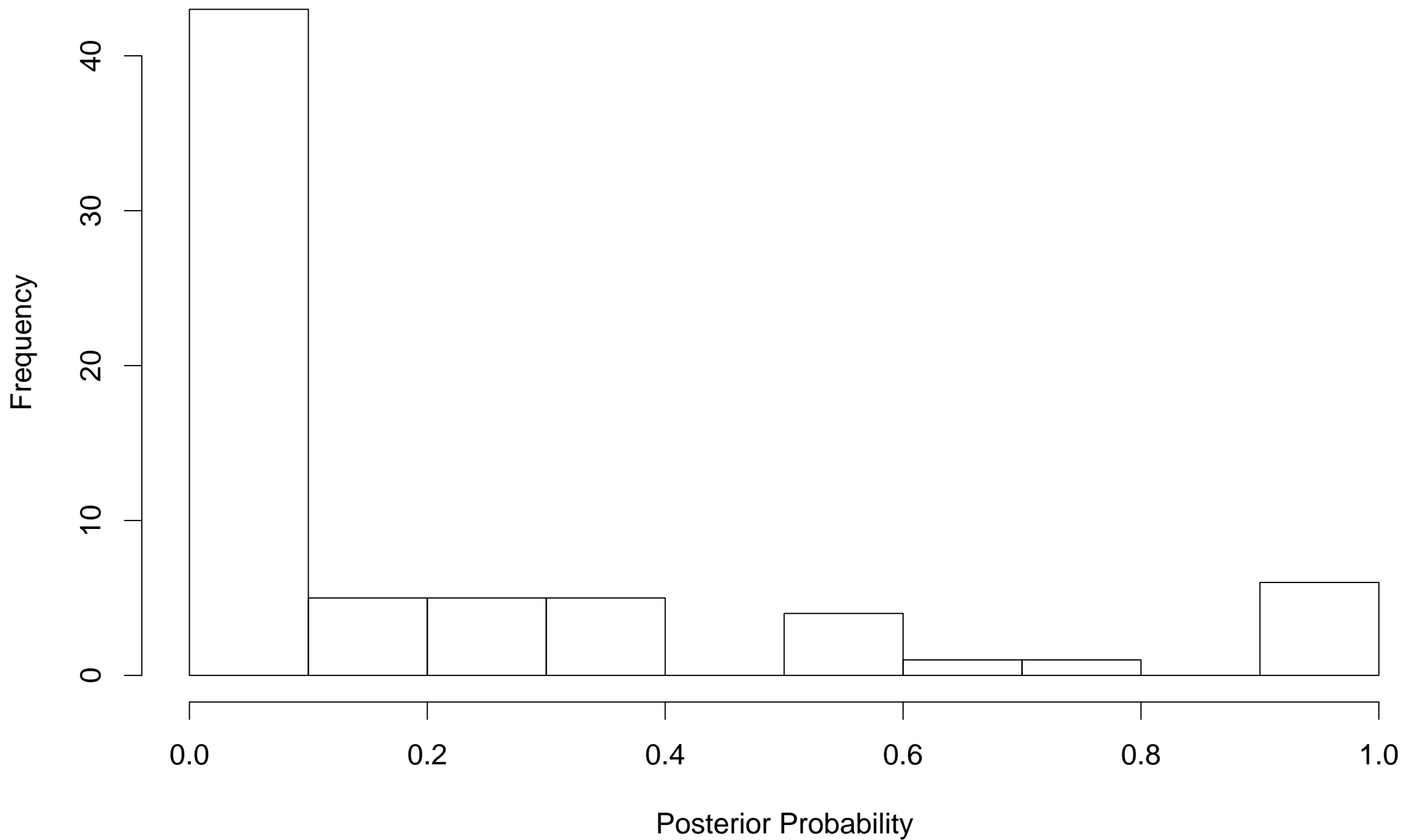

Supplement: Additional File 16 — Histogram of the posterior probabilities for the small group of the TOR2 signaling mixture model. The pdf-file contains a figure showing the distribution of posterior probabilities for the small group of tor2 genetic interaction partners. High posteriors mean high likelihood for the respective genes of being involved in TOR2 signaling. [file 1752-0509-2-3-S16.pdf]

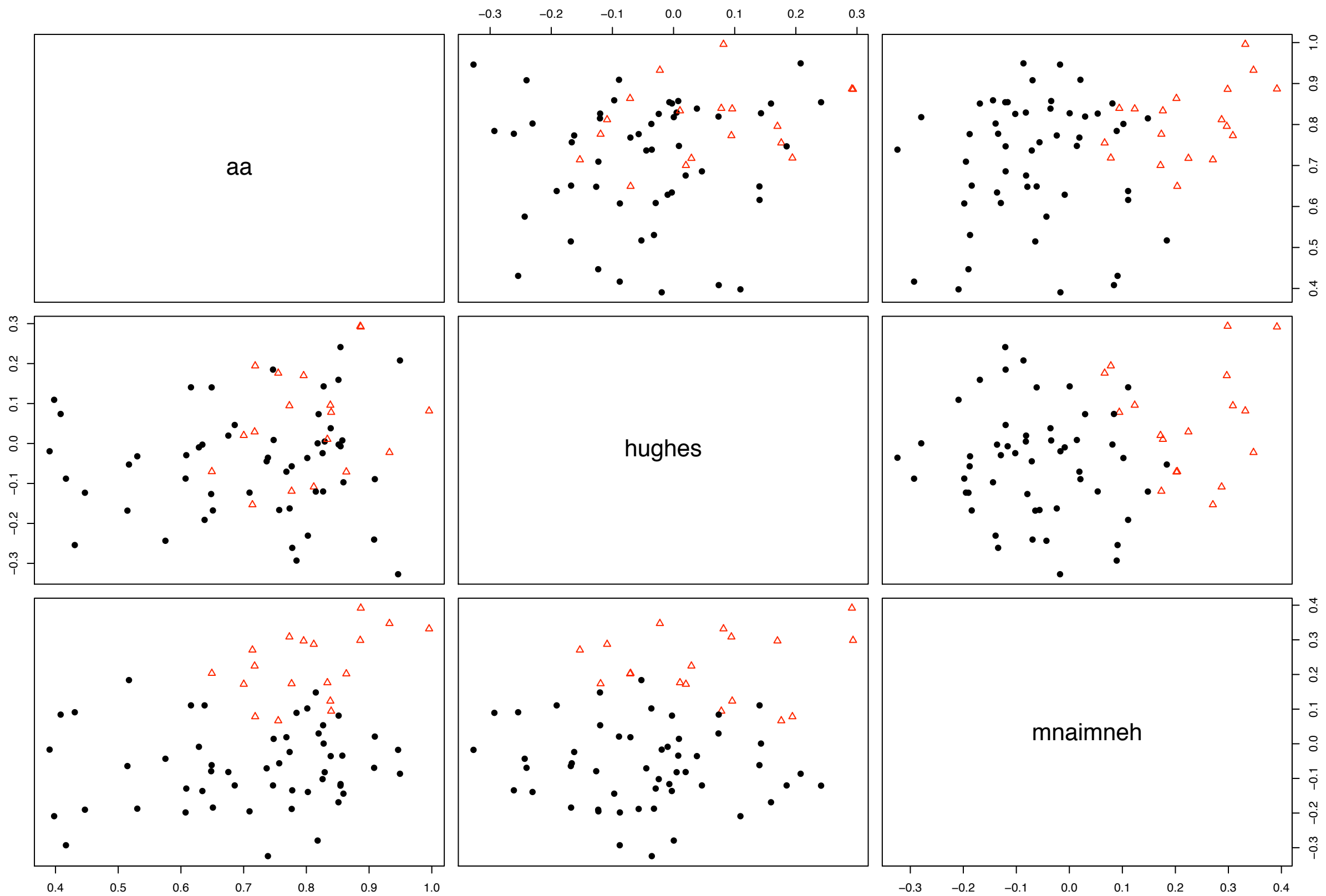

Supplement: Additional File 18 — Best subset of features for TOR2 signaling. The pdf-file shows the scatterplot for the best subset of features for the TOR2 signaling mixture model {aa, hughes, mnaimneh} with a cutoff of 0.29. Target genes belonging to the small group are marked by red triangles, the big group is marked in black. The members of the small group all share very high values for amino acid correlation (aa) and intermediate to high values of correlation in gene expression data with respect to tor2 (hughes and mnaimneh). [file 1752-0509-2-3-S18.pdf]
